# A novel biweekly multidrug regimen of gemcitabine, oxaliplatin, 5-fluorouracil (5-FU), and folinic acid (FA) in pretreated patients with advanced colorectal carcinoma

**DOI:** 10.1038/sj.bjc.6601783

**Published:** 2004-04-13

**Authors:** P Correale, S Messinese, M Caraglia, S Marsili, A Piccolomini, R Petrioli, F Ceciarini, L Micheli, C Nencini, A Neri, G Vuolo, A Guarnieri, A Abbruzzese, S D Prete, G Giorgi, G Francini

**Affiliations:** 1 Section of Oncology, Human Pathology and Oncology Department, University of Siena School of Medicine, Viale Bracci 11, Siena 53100, Italy; 2 Oncology Operative Unit, Frattamaggiore Hospital, Italy; 3 Surgical Science Department, University of Siena School of Medicine, Italy; 4 ‘Giorgio Segre’ Pharmacology Department, University of Siena School of Medicine, Italy; 5 Biochemistry Section, Second University of Naples, Naples, Italy

**Keywords:** colorectal cancer, gemcitabine, oxaliplatin, 5-fluorouracil, folinic acid

## Abstract

Previous results suggest that GEM affects 5-fluorouracil (5-FU) metabolism and pharmacokinetics in cancer patients, while combined with oxaliplatin, levo-folinic acid, and 5-FU (GOLF regimen), at doses achievable in cancer patients, determines high cytotoxic and proapoptotic antitumour activity in colon cancer cells *in vitro*. On these bases we designed a phase I–II clinical trial testing the GOLF regimen in patients with metastatic colorectal carcinoma, who had received at least a prior line of chemotherapy. In total, 29 patients (20 males and nine females) enrolled in the study received every 2 weeks, gemcitabine (patients #1–3 received 600 mg m^−2^; patients # 4–6 received 850 mg m^−2^; while patients # 7–29 received 1000 mg m^−2^) on the day 1, levo-folinic acid (100 mg m^−2^) on the days 1 and 2; 5-fluorouracil (400 mg m^−2^) in bolus injection, followed by a 22-h continuous infusion (800 mg m^−2^) on the days 1 and 2, and oxaliplatin (85 mg m^−2^), 6 h after the 5-FU bolus on day 2. The most frequent side effect was grade I–II haematological toxicity. In total, 28 patients were evaluable for response: three achieved a complete response, nine a partial response, 10 had a stable disease, and six progressed. The average time to progression and overall survival of the patients was, respectively, 7.26 and 22 months. Our GOLF combination is well tolerated and seems promising for the treatment of advanced colorectal cancer.

Colorectal carcinoma is the second leading cause of cancer death in the Western countries and almost 50% of these patients die because of problems related to disease progression. Although a higher rate of response has been achieved with the newest polychemotherapy regimens, patients with metastatic colorectal cancer still have poor prognosis with a dismal 5-year survival rate (Gill *et al*, 2003).

5-Fluorouracil (5-FU) alone or in combination with biomodulators, such as folinic acid (FA), levamisol, interferon alpha, has long been the only systemic treatment in adjuvant setting and advanced stage of disease (Francini *et al*, 1994; Grem, 1996; O’Connell, 1998; Von Hoff, 1998; Skibber, 2001), but new drugs such as CPT-11 and the novel platinum derivative oxaliplatin have recently been successfully combined with 5-FU in different administration schedules and dosages (Skibber, 2001; Von Hoff, 1998). The combination of oxaliplatin with FA and infusional 5-FU (FUFA) is currently being investigated by the American Food and Drug Administration (FDA) in the first-line treatment for advanced colorectal carcinoma. This new generation of drugs has been evaluated in a number of clinical trials and has certainly improved clinical response rates and survival; unfortunately, the average overall survival still remain within the 20 months also in the most optimistic studies (Grothey and Schmoll, 2001; Kohne, 2003; Goldberg *et al*, 2004; Tournigand *et al*, 2004).

The results of these trials, therefore, justify the continuing search for new drugs or more active drug combinations. It has been shown that the difluorinated analogue of deoxycytidine known as gemcitabine (GEM) (difluoro-2′,2′-deoxycytidine) synergistically interacts with 5-FU in terms of antitumour activity in colon carcinoma cells *in vitro* (Correale *et al*, 1999). The combination of these two drugs administered by using different schedules and dosages has also showed a significant antitumour activity in patients with a different gastroenteric malignancies (Berlin *et al*, 1998–1999; Schulz *et al*, 1998; Correale *et al*, 1999; Hidalgo *et al*, 1999) including colo-rectal carcinoma (Madajewicz *et al*, 2000). Previous studies of our group have in addition shown a supra-additive cytotoxic activity and apoptosis-inducing capacity of GEM combined with oxaliplatin and FUFA (GOLF) in colon cancer cell lines *in vitro* (Correale *et al*, 2002 and unpublished results). On these bases, we have designed a phase I–II clinical trial aimed to investigate in patients with advanced colorectal carcinoma who had received at least a line of chemotherapy, the toxicity and the antitumour activity of a new polychemotherapy regimen composed by GEM plus oxaliplatin and FUFA administered using the schedule suggested by de Gramont *et al* (1997), (1998)) (FOLFOX-4).

## MATERIALS AND METHODS

### Eligibility criteria

The inclusion criteria required a histological diagnosis of colorectal carcinoma, a Eastern Cooperative Oncology Group (ECOG) performance status ⩽2, a life expectancy >3 months, normal renal and liver function, a white blood cell count >2500 cells mm^−3^, haemoglobin >9 g dl^−1^, platelet cell count >100 000 cells mm^−3^, a cardiac ejection fraction >46%, and a normal electrocardiogram. The exclusion criteria included heart, liver or kidney failure, cardiac valvular and wall motion abnormalities, central nervous system involvement, second tumours, active infectious disease, or a history of cardiovascular disease. The study was approved by our local (University) ethics Committee, and respected the guidelines for good clinical practice. All of the patients gave their written informed consent.

The trial was designed to test the hypothesis that the GOLF combination is tolerated and active in the treatment of colorectal carcinoma. A minimum of 25 patients was required in order to maintain an alpha and beta error of, respectively, 0.05 and 0.2. All of the patients had previously received at least one line of chemotherapy.

### Clinical assessments

A complete history was taken, and a physical examination, complete blood count, and serum chemistry evaluation were performed at the baseline. Complete disease staging by chest X-ray, chest and abdominal computed tomography, and liver and pelvis ultrasonography was undertaken at the baseline and after four, six and 12 treatment cycles. All of the eligible patients were evaluated for survival and toxicity, and all of the patients were considered evaluable for response. The patients continued the treatment until the onset of unacceptable toxicity or disease progression for a maximum of 18 cycles (9 months of treatment).

### Response criteria

Response and toxicity were assessed according to standard World Health Organization (1979) criteria.

## RESULTS

### Clinical trial design, patient characteristics, and treatment schedule

This clinical trial involved patients with advanced colorectal carcinoma, who had received at least one line of chemotherapy, and was divided into two phases: the first was designed to test the toxicity of the GOLF combination, and the second its antitumour activity. The trial protocol foresaw early trial discontinuation in the case of unacceptable toxicity or if no clinical response was observed in the first 12 patients. In the first phase of the trial, three groups of three patients each were administered standard doses of oxaliplatin, FA, and 5-FU using the schedule proposed by de Gramont *et al* (1997), (1998) and escalating doses of GEM. The schedule of treatment was designed taking in consideration the results of previous preclinical (Correale *et al*, 1999, 2002), and pharmacokinetics (Correale *et al*, 2003) studies; thus all patients received GEM, at different doses (see below) in a 30-min intravenous (i.v.) infusion on day 1, before any other drug; subsequently, they received levo-FA 100 mg m^−2^ in a 30-min i.v. infusion on days 1 and 2; 5-FU 400 mg m^−2^ in a 30-min i.v. infusion, followed by a 22-h continuous infusion (800 mg m^−2^) on days 1 and 2; and oxaliplatin 85 mg m^−2^ in a 2–4 h i.v. infusion before the second FUFA administration on day 2. The treatment was repeated every 15 days.

A first group of three patients received GEM at the dosage of 600 mg m^−2^ (patients #1–3), a second group of three patients (# 4–6) received a dose of 850 mg m^−2^, and a third group (# 7–9) received a dose of 1000 mg m^−2^. Previous studies, investigating the two drug combination of GEM with 5-FA or with oxaliplatin in different gastroenteric malignancies, suggest that the limiting dose of GEM is 1000 mg m^−2^, thus in our study when this level of GEM dose was reached in combination with oxaliplatin+FUFA without the occurrence of significant side effects, all of the subsequently enrolled patients, belonging to the phase II (patients #10–29), received this dose of drug.

The trial population consisted of 29 patients (20 males and nine females), with an average age of 67.7 year (range 42–77). They all had a histological diagnosis of colorectal carcinoma, had undergone radical resection of the primary tumour, and were in advanced stage of disease at the time of the enrolment. Their ECOG performance status ranged from 0 and 2, and their life expectancy was longer than 3 months. In total, 20 patients had previously received one line of treatment, seven had received two lines, and two had received three lines (Table 1

**Table 1:** Clinical trial design

**Characteristics**	**Number of patients**
Patients evaluable for response	28
Patients evaluable for toxicity	29
*Sex*	
Male	20
Female	9
Performance status (ECOG)	0–2
*Primary site*	
Sigma and left colon	21
Right colon	8
Stage IV	29 (100%)
*Disease extension*	
(A) Liver	24
(B) No liver (bone+lung+peritoneum+ovary)	5
*Previous treatments*	
Surgery	27
One line of chemotherapy	20
Two or more lines of chemotherapy	9
FUFA	23
IFL	7
FOLFOX-4	3
Raltitrexed	1
Intra-hepatic mytomicin C infusion	3

The median age of the patients was 67.7 year (range 42–77).

).

### Toxicity profile

A total of *296* chemotherapy cycles were administered (a median range of *10* cycles per patient: range 6–18 cycles). No treatment delay due to neutropenia or thrombocytopenia was required. Moderate and severe asthenia was reported during the second week of treatment. One patient experienced grade 3–4 gastroenteric toxicity with diarrhoea, and few cases of moderate mucositis and vomiting were recorded after oxaliplatin administration in the first cycle. Grade 1–2 haematological toxicity with moderate anaemia, neutropenia, and thrombocytopenia was the most common adverse event. No persistent thrombocytopenia was observed after multiple cycles of chemotherapy. None of these patients experienced increased creatinine or blood urea nitrogen, or hypotension. Three patients manifested reversible mucosal toxicity, nausea/vomiting and diarrhoea, and two reversible grade 1–2 peripheral neurotoxicity. Three patients developed hypersensitivity to oxaliplatin, and one of them had to be withdrawn from the trial. There were no toxicity-related dose reductions or treatment delays. Two days after the third cycle of treatment, one patient developed a sudden gastric haemorrhage with hypovolemic shock and eventually died for its consequences. Two patients experienced reversible atrial fibrillation, one during oxaliplatin administration and the other one 24 h after the end of the treatment. The first one of these two was the same patient who showed hypersensitivity to oxaliplatin, while the second one had this episode at the end of the 12th cycle of treatment (Table 2

**Table 2:** World Health Organization (WHO) toxicities (number of patients=29)

	**Number of events (%)**	**Grade 1 *N* (%)**	**Grade 2 *N* (%)**	**Grade 3 *N* (%)**	**Grade 4 *N* (%)**
*Hematological*	11 (37.9%)				
Anaemia	4 (13.8%)	2 (6.9%)	2 (6.9%)		
Neutropenia	6 (20.6%)	1 (3.45%)	2 (6.9%)	3 (10.3%)	
Thrombocytopenia	1 (3.45%)				1 (3.45%)
					
*Gastroenteric toxicity*	7 (24.1%)				
Nausea/vomiting	2 (6.9%)	1 (3.45%)			1 (3.45%)
Diarrhoea	2 (6.9%)	1 (3.45%)			1 (3.45%)
Mucositis	3 (10.3%)	2 (6.9%)			1 (3.45%)
					
Transaminase elevation	None				
Fever	8 (27.6%)	3 (10.3%)	5 (17.2%)		
Asthenia	2 (6.9%)			1 (3.45%)	1 (3.45%)
Anorexia	None				
Neurological toxicity	2		2 (6.9%)		
Arrythmia	2		2(6.9%)		
Hypersensitivity	3 (10.3%)	1 (3.45%)	1 (3.45%)	1 (3.45%)	

A total of *296* chemotherapy cycles were administered (a median range of 10 cycles per patient).

).

### Response and survival

In total, 28 patients were evaluable for response: three achieved a complete response, nine a partial response, 10 had a stable disease, and six progressed. One patient was not evaluable for response due to early toxicity related death. The time to progression and the overall survival of each patient are reported in Table 3

**Table 3:** Clinical response of advanced colorectal adenocarcinoma patients to gemcitabine, oxaliplatin, and infusional levo-folinic acid and 5-fluorouracil (GOLF) multidrug chemotherapy

**Number**	**PD**	**CR**	**PR**	**SD**	**NE**
289	6	3	9	10	1
100%	20.7%	10.3%	31.2%	34.4%	3.4%

PD=progressive disease; CR=complete response; PR=partial response; SD=stable disease; NE=nonevaluable for response for early death. The time to progression was 7.26 months (95% of CI 6.88–12.62). The overall survival was 22 months (95% CI 12.6–26.5).

. Although the follow-up is too short to provide definitive results, the clinical response rate, the time to progression (7.26 months, 95% CI, I6–11 months), and the overall survival (22 months) seem to be interesting, especially when compared with those obtained with the most widely used second-line of treatments (Table 3).

## DISCUSSION

Since its introduction by Heidelberger *et al* in 1957, 5-FU has been one of the main components of treatments for the most common malignancies. It is a prodrug that requires conversion by cancer cells in the active metabolites, 5-fluorodeoxyuridine monophosphate (5FdUMP) and 5-fluorouridine triphosphate (5FUTP) (Chu *et al*, 2001). The most optimistic studies testing 5-FU for first-line in colo-rectal carcinoma reported overall response rate lower than 15%, with a median survival of 12–18 months. Clinical evidence suggests that the use of protracted intravenous infusion of 5-FU may be more efficacious and better tolerated than bolus dosing (Cancer, 1998), and that modulation of 5-FU may lead to higher response rates (Poon *et al*, 1989, 1991). Oxaliplatin is a novel diaminocyclohexane platinum agent that mainly acts by causing inter- and intrastrand DNA crosslinks (Mathe *et al*, 1989; Soulie *et al*, 1997; Wiseman *et al*, 1999; Culy *et al*, 2000). It has a wide range of antitumour activity and promising effects in the treatment of colorectal carcinoma (de Gramont *et al*, 1997, 1998; Wiseman *et al*, 1999; Fischel *et al*, 1998, 2002). The potential benefit of oxaliplatin plus FUFA (FOLFOX) over FUFA alone has been largely demonstrated (de Gramont *et al*, 1998; Giacchetti *et al*, 2000; Goldberg *et al*, 2002). More recently, based on the results of a NCI clinical trial showing its superiority (in term of toleration, objective response rate, time to progression, and overall survival) over the CPT-11, FA, and 5-FU combination (IFL), the US FDA is considering to expand the current indication of Oxali-FUFA (in accord with the FOLFOX-4 schedule proposed by de Gramont) to the first-line treatment for advanced colorectal carcinoma (Goldberg *et al*, 2002).

GEM is a *difluoro-2′,2′-deoxycytidine* that requires activation through the synthesis of its phosphorylated metabolites (difluoro deoxycytidine-diphosphate and triphosphate) in order to induce DNA damage, to block the DNA repair system, and to affect the deoxy-nucleotide synthesis (Plunkett *et al*, 1996; Chu *et al*, 2001).

On the basis of the first enthusiastic results of the early preclinical and clinical studies mainly concentrated on NSCLC and pancreas carcinoma, GEM moved very rapidly from phase I/II to phase III trials (especially in combination with other established anticancer drugs) leaving partially unexplored its activity in several common cancers, such as colon and gastric carcinoma (Plunkett *et al*, 1996; Grem *et al*, 1999; Heinemann, 2001). With the exception of one abstract published at the ASCO meeting in the far 1992, single agent GEM antitumour activity in patients with colorectal carcinoma has not been directly demonstrated. On the other hand, previous studies have shown that GEM induces major alterations in the pharmacodynamics, pharmacokinetics, and antitumour activity of 5-FU that may lead to positive antitumour results also in colon carcinoma patients (Berlin *et al*, 1998–1999; Schulz *et al*, 1998; Correale *et al*, 1999, 2000b, 2000a, 2003; Poplin *et al*, 1999; Madajewicz *et al*, 2000; Peters *et al*, 2000).

In more recent preclinical studies we also found that GEM, used at concentrations potentially achievable in patients, is able to enhance the cytotoxic and proapoptotic activity of oxaliplatin and FUFA combination (Correale *et al*, 2000b, 2000a, 2002, and unpublished data). The antitumour effects of the GOLF regimen was far greater than those induced by the combinations of GEM/5-FU, oxaliplatin/GEM, or oxaliplatin/5-FU (unpublished results). In line with these results, other groups also showed that oxaliplatin can have supra-additive antitumour activity when used in combination with GEM (Faivre *et al*, 1999; Mani *et al*, 2000; Raymond *et al*, 2001). This effect, found in various tumour cell lines *in vitro*, has been explained on the ground that GEM blocks the nuclear mechanisms used by cancer cells to remove oxaliplatin adducts and to allow DNA repair (Faivre *et al*, 1999; Mani *et al*, 2000; Raymond *et al*, 2001) with consequent induction of apoptosis. In the present trial, the combined administration of GEM, oxaliplatin, and FUFA is well tolerated and seems to have a high degree of antitumour activity. With the exception of GEM, all of the drugs could be used at full doses without the occurrence of significant side effects, and their combination led to a high rate of objective responses (CR+PR) (42%) and stable diseases (34.5%) with a time to progression and an average survival, respectively, of 7.26 and 22 months. These results acquire greater importance if we consider that the majority of the patients had previously received at least one first-line of 5-FU-based treatment, and that the particular design of the trial meant that the first six patients received suboptimal doses of GEM and should, therefore, not be considered in term of clinical response. In conclusion, the GOLF combination seems promising for the treatment of advanced colorectal carcinoma. It deserves to be studied in larger phase III clinical trials in order to investigate its real impact on the survival of these patients compared with the standard FOLFOX-4 regimen.
